# Combined Direct and Indirect CT Venography (Combined CTV) in Detecting Lower Extremity Deep Vein Thrombosis

**DOI:** 10.1097/MD.0000000000003010

**Published:** 2016-03-18

**Authors:** Wan-Yin Shi, Li-Wei Wang, Shao-Juan Wang, Xin-Dao Yin, Jian-Ping Gu

**Affiliations:** From the Department of Interventional Radiology (W-YS, J-PG); The Medical Imaging Center, Nanjing First Hospital, Nanjing Medical University (L-WW, X-DY); and The department of diagnostic radiology, Jiangsu Hospital of Traditional Chinese Medicine (S-JW), Nanjing, China.

## Abstract

This study aimed to evaluate the diagnostic accuracy of combined direct and indirect CT venography (combined CTV) in the detection of lower extremity deep vein thrombosis (LEDVT).

The institutional review board approved the study protocol, and patients or qualifying family members provided informed consent. A total of 96 consecutive patients undergoing combined CTV were prospectively enrolled. A combined examination with digital subtraction angiography (DSA) plus duplex ultrasonography (US) was used as the criterion standard. Three observers were blinded to clinical, DSA, and US results, and they independently analyzed all combined CTV datasets. Interobserver agreement was expressed in terms of the Cohen k value for categorical variables. Accuracy, sensitivity, specificity, positive predictive value (PPV), and negative predictive value (NPV) of combined CTV in the detection of LEDVT were determined by using patient- and location-based evaluations.

Of the 96 patients, DSA plus US revealed LEDVT in 125 segmental veins in 63 patients. Patient-based evaluation with combined CTV yielded an accuracy of 96.9% to 97.9%, a sensitivity of 95.2% to 96.8%, a specificity of 100% to 100%, a PPV of 100% to 100%, and an NPV of 91.7% to 94.3% in the detection of LEDVT. Location-based evaluation yielded similar results. Through combined direct and indirect CTV, patients obtained a combined CT angiogram on the diseased limb and an indirect CT angiogram on the opposite side. The image quality of combined CTV was superior to an indirect venogram.

Combined CTV shows promising diagnostic accuracy in the detection of LEDVT with 3-dimensional modeling of the lower limb venous system.

## INTRODUCTION

Deep vein thrombosis (DVT), together with pulmonary embolism, has long been considered as a single disease, namely venous thromboembolism. DVT is a potentially life-threatening disease with >200,000 first lifetime cases reported each year in the United States.^1–3^ DVT occurs most often in the leg, which is named as lower extremity DVT (LEDVT).^4^ LEDVT is of clinical significance because it can not only produce chronic venous insufficiency in the diseased leg, but also leads to pulmonary embolism and subsequent chronic thromboembolic pulmonary hypertension. Therefore, all patients with suspected LEDVT should be investigated until a definitive diagnosis is reached.

In patients with suspected LEDVT, venous ultrasound is the option of choice. Another potential diagnostic tool is contrast enhanced or non-enhanced magnetic resonance imaging, which could be useful in patients with contraindications to CT pulmonary angiography (contrast-induced nephropathy, allergy) or in pregnant women. However, the overall sensitivity of this modality in detecting DVT is limited.^5–7^ Previous studies have assessed the merits of computed tomography venography (CTV) in the investigation of LEDVT, as well as pulmonary embolism, with a sensitivity of 89% to 100% and specificity of 94% to 100%.^5–9^ The techniques used in CTV involve indirect and direct venography of the lower extremities.^5,10,11^ In clinical practice, indirect CTV is the most frequently used technique and usually adds to CT pulmonary angiography. Both of these techniques are a package protocol so that identification of pulmonary embolism and/or LEDVT can be processed in batches.^5,11^ The main disadvantage of indirect CTV is poor attenuation of deep veins that is usually associated with individual differences in circulation time of contrast medium reaching the limbs. Therefore, imaging quality might be compromised because of the lower density contrast between background and veins. Three-dimensional (3D) images produced by direct CTV are better than those by indirect CTV, but the existence of artifacts may result in false-positive diagnosis,^5,10,11^ especially in the case of small, localized thrombus.

In the present study, we introduced a new technique called combined direct and indirect CTV (combined CTV), with the combination of direct and indirect CTV together. With the combination of direct and indirect CTV, diseased veins can be displayed with systematic plus local enhancement. We supposed that this combination and dual effects of enhancement can theoretically overcome the disadvantages that belong to either direct or indirect CTV.^5,10,11^ Diseased veins can be displayed as 3D images with high quality, which can help in pre-operational planning. Systematic enhancement of diseased veins can avoid some artifacts related to direct CTV (eg, “injection loss of some veins”).^5,10,11^ The combined CTV did not expose the patients to more radiation harm compared to direct or indirect CTV because of no more scanning sequences added. In the present study, we aimed to determine the performance of this technique on the investigation of LEDVT.

## MATERIALS AND METHODS

This study protocol was approved by the institutional review board and the ethical committee, Nanjing First Hospital, Nanjing Medical University. Informed consent was obtained from all of the patients or immediate family members.

### Patients

Between January 2011and June 2013, 103 patients who were suspected as having LEDVT were enrolled in this study. Seven patients were excluded because of renal dysfunction (n = 5) and heart failure (n = 2). Finally, 96 consecutive patients underwent combined CTV of the lower extremities in our tertiary hospital. The onset symptoms included lower extremity swelling in 85 patients, pain in 76, and skin pigmentation in 23. The median duration of symptoms in patients with acute DVT was 7 days, whereas that in the negative control group was 3.5 years. All of the patients underwent ascending venography and duplex ultrasonography (US) after combined CTV was performed. For patients who intended to undergo endovascular treatment, transcatheter venography was performed for decisive diagnosis. The combination of US and digital subtraction angiography (DSA) was used as the criterion standard, and showed acute LEDVT in 63 patients and other venous diseases in 33 patients. Therefore, 63 patients with LEDVT and 33 patients with no DVT (ie, negative control subjects), were included in the final analysis. The study group comprised 51 men and 45 women, with a mean age of 53.01 ± 11.63 years (24–87 years).

### Combined CTV Protocol of LEDVT

**FIGURE 1:**
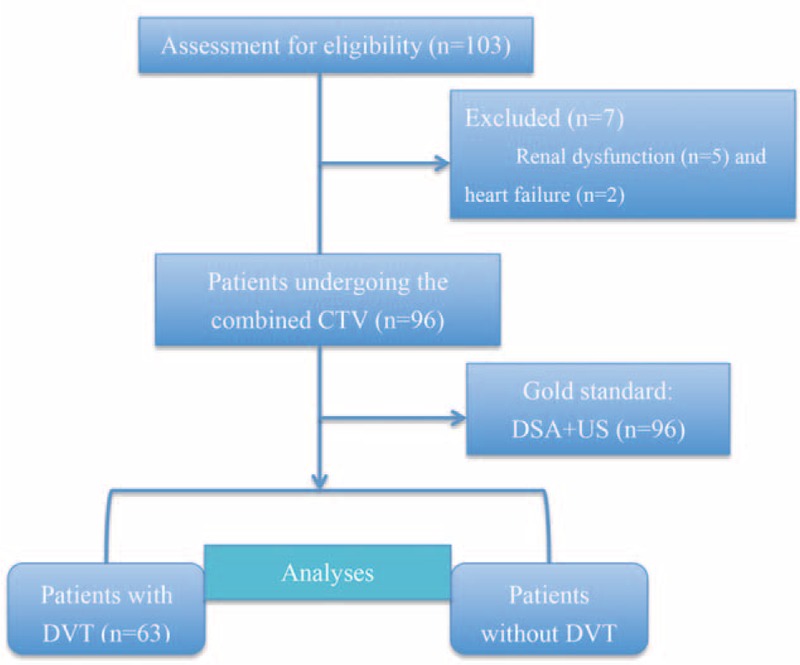
Flow diagram of patients who were suspected of having DVT and who underwent combined direct and indirect CTV (combined CTV). CTV = computed tomography venography, DVT = deep vein thrombosis, DSA = digital subtraction angiography, MTS = May–Thurner syndrome, PTS = post-thrombotic syndrome.

All CTVs were performed with a 128-slice second-generation dual-source CT scanner (Somatom Definition Flash, Siemens, Germany). We used a dual-energy scanning method with non-electrocardiogram-gated acquisition to obtain images from the level of the first lumbar vertebra to the toes for all patients. Moreover, non-contrast scanning of the same area was first performed as a baseline before CTV. A flow chart of the patients is shown in Figure 1.

The term “combined direct and indirect CTV (combined CTV)” in the present study refers to a scanning technique that packaged direct and indirect CTV together. Direct CTV is commonly attained by injection of contrast medium via the diseased dorsal vein followed by scanning of diseased limbs.^5,10^ Indirect CTV is usually performed post-injection of contrast medium via the cubital vein. In clinical practice, the accomplishment of combined CTV requires 3 steps to be performed in succession. First, 80 mL of contrast medium (ioversol 320 mg/mL; Jiangsu Henrui Medicine, Co, Ltd, Jiangsu, China) via the right cubital vein was injected at a rate of 4 mL/s. A volume of 60 mL suspension consisting of 12 mL ioversol and 48 mL normal saline was then injected 1.5 minutes after the first injection, via the diseased dorsal vein at a rate of 1.5 mL/s. A tourniquet was placed around the ankle during the second injection so that the contrast medium was forced to enter the deep veins. During this process, 2 sets of binocular high-pressure syringes (Stellant, Medrad Inc, Indianola, PA) were equipped, one for the first and the other for the second injection of contrast medium. Therefore, a total of 92-mL contrast medium was needed for use in a unilateral extremity. Finally, 1 minute after the second injection, CT scanning of the aforementioned area (from the level of the first lumbar vertebra to the toes) was commenced.

A dual-energy scanning protocol was uniformly applied for all patients, with the following parameters: voltage, 100 kV and Sn 140 kV with a voltage ratio of 0.5; current, 210 to 160 mAs, automatically tracked with Care Dose 4D; collimator width, 128 × 0.60 mm; pitch, 0.7; tube rotation time, 0.33 s per circle; field of view, 35 to 45 mm; and original acquisition thickness, 0.60 mm.

### Post-processing Techniques

Thin-section images (thickness of 0.75 mm and interval of 0.7 mm) were automatically reconstructed and saved on a workstation (Somatom Definition Flash workstation, Siemens Healthcare, Forchheim, Germany). We used various techniques, including multiple planar reconstruction, curved planar reconstruction, volume rendering, and maximum intensity projection to reconstruct 3D images of vessels at arbitrary angles and orientations. In the reconstructed images, we were also able to adjust the threshold of window level and width so that the optimal images could be displayed and stored.

### Digital Subtraction Angiography for LEDVT

Ascending venography was performed for all of the patients 1 day post-CTV. The patients were placed on a DSA table (Artis Zee Ceiling, Siemens Healthcare) in the supine position. A tourniquet was placed around the ankle to prevent flow through the superficial venous system, but to force the contrast medium to enter only the deep veins. A superficial vein on the dorsum of the foot was then accessed by a 21-G needle, which was connected to a high-pressure syringe. DSA imaging was acquired in the caudocranial direction, from the foot to the lower segment of the inferior vena cava. This usually required 4 successive and separated sessions of acquisition. During each of the 3 sessions of acquisition from the foot to the groin, 10-mL ioversol was injected at a rate of 1 mL/s. During the fourth session aiming at visualization of the veins above the groin, 20-mL ioversol was injected at a rate of 1.5 to 2.0 mL/s. Therefore, a total of 50-mL contrast medium was almost always used in venography of a unilateral lower extremity. Fluoroscopic imaging with the patient in various positions rarely required additional consumption of contrast medium. The Valsalva maneuver was added to observe competent venous valves and venous reflux.

### Duplex Ultrasonography for LEDVT

The procedure for US was primarily based on examination of all patients using the GE LOGIO 9 (GE Healthcare, USA) or Philips IU22 (Philips, Amsterdam, Netherlands) using high-frequency or multifrequency probes. Deep veins were examined from the common femoral vein up to the distal posterior tibial vein. A lack of compressibility of a venous segment under the ultrasound probe was considered as diagnostic of DVT. A Doppler flow study was used to diagnose iliac vein thrombosis, particularly where veins were not compressible.

### Interpretation of Images

Three experienced observers, all blinded to clinical, DSA, and US results, and independent of each other, interpreted all CT and CTV datasets on an offline workstation. These observers were allowed to adjust the appropriate threshold of window width and level and rotate 3D images arbitrarily for comprehensive visualization. Cases with detection of ≥1 segmental venous thrombosis were considered positive, regardless of whether May–Thurner syndrome, post-thrombotic syndrome, or any other venous diseases coexisted. All other cases were considered negative. Locations of thrombosis were classified as 3 segments: the calf veins; the popliteal and femoral veins; and the iliac vein.

### Statistical Analysis

Categorical demographic and basic characteristic variables are shown as numbers and percentages, whereas continuous variables are shown as means ± standard deviations. The results from the individual readers were analyzed separately. Interobserver agreement was expressed in terms of the Cohen *k* value for categorical variables. Descriptive statistical analyses were performed on 2 levels: patient-by-patient (no or any DVT per patient), and location-by-location. The diagnostic performance parameters of combined CTV in the diagnosis of DVT relative to DSA and US as the reference standards are shown in terms of overall accuracy, sensitivity, specificity, positive predictive value (PPV), negative predictive value (NPV), and the area under the receiver-operating characteristic curve.

## RESULTS

The demographic and clinical data of the 96 patients are shown in Table 1.

**TABLE 1:**
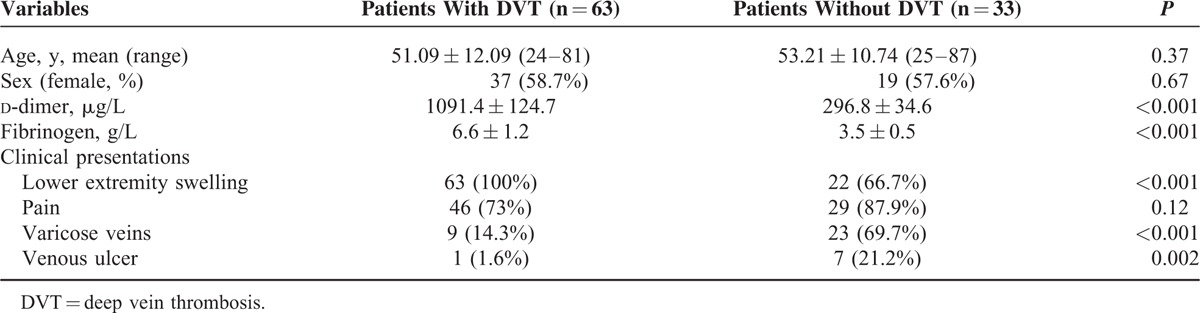
Demographic and Clinical Data of 96 Patients

DSA plus US images showed 125 segmental thrombosis in 78 lower limbs in 63 patients. Forty-eight patients had unilateral LEDVT and 15 patients had bilateral LEDVT. Overall, 38 segmental DVTs were located at the iliac veins, whereas 71 and 16 DVTs were located at the popliteal and femoral veins and calf veins, respectively. Thirty-three patients in the negative control group received examinations (combined CTV, as well as DSA and US) on 45 limbs, just including 135 segmental veins. These examinations showed no evidence of acute LEDVT, but May–Thurner syndrome was observed in 21 iliac veins, superficial varices in 13 limbs, and post-thrombotic syndrome in 11 limbs.

During CT scanning, motion artifacts occurred in 3 patients, which initially led to uninterpretable image quality. Patients were aware of potential impairment to renal function that was directly related to increments of contrast medium used in the short term, and they were asked to undergo a second examination 12 hours later. The image quality was subsequently improved so that the patients were eligible for diagnosis. The primary technical success rate was 96.9% (93/99) and the secondary success rate was 100%. The mean radiation dose of combined CTV was 682.8 ± 136.2 mGycm (range: 501–954 mGycm). No complications related to radiation exposure or contrast medium were reported.

We found that combined CTV with volume rendering images alone enabled us to confirm the diagnosis in only 107 of 125 segmental veins and that the remaining 18 lesions were not clearly delineated on the volume-rendering images. This was more frequently encountered at the popliteal and calf veins. The final evaluation for LEDVT in this study was made according to the findings of combined CTV with multiple planar reconstruction, curved planar reconstruction, and maximum intensity projection, as well as with cross-sectional images.

The diagnostic performance of combined CTV in the detection of LEDVT compared with DSA in patient- and location-based evaluation is shown in Tables 2 and 3. Compared with DSA plus US, observers A and C missed 2 segmental DVTs in 2 patients who had combined CTV, and observer B missed 3 segmental DVTs in 3 patients (Table 4). No false-positive DVTs were observed either by observer A, B, or C. The sensitivity, specificity, NPV, PPV, and accuracy of patient-based combined CTV in the detection of LEDVT was 98.1%, 100%, 93.4%, 100%, and 97.6%, respectively. The sensitivity, specificity, NPV, PPV, and accuracy of US that was used alone to detect LEDVT was 93.7%, 96.7%, 88.9%, 98.3%, and 94.8%, respectively. However, there was no significant difference between combined CTV and US in the detection of LEDVT in terms of diagnostic accuracy (*P* = 0.17). In location-based evaluation, the sensitivity, specificity, NPV, PPV, and accuracy of combined CTV in the detection of iliac vein thrombosis were all 100%, whereas those of US were 94.7%, 96.5%, 97.6%, 92.3%, and 95.9%, respectively.

**TABLE 2:**
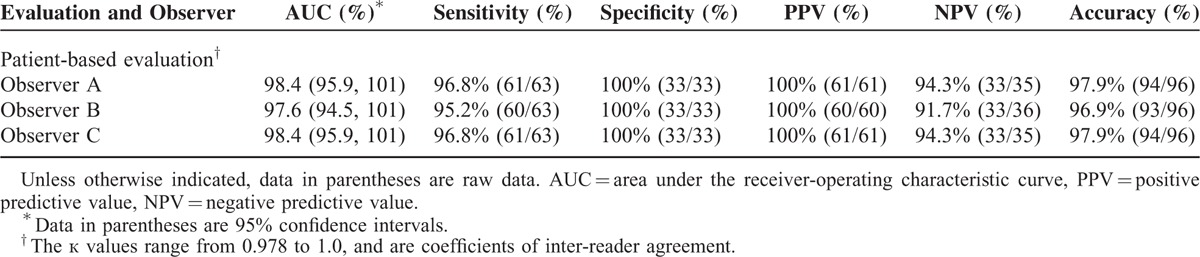
Diagnostic Performance of Combined Direct and Indirect CTV in Patient-based Evaluation

**TABLE 3:**
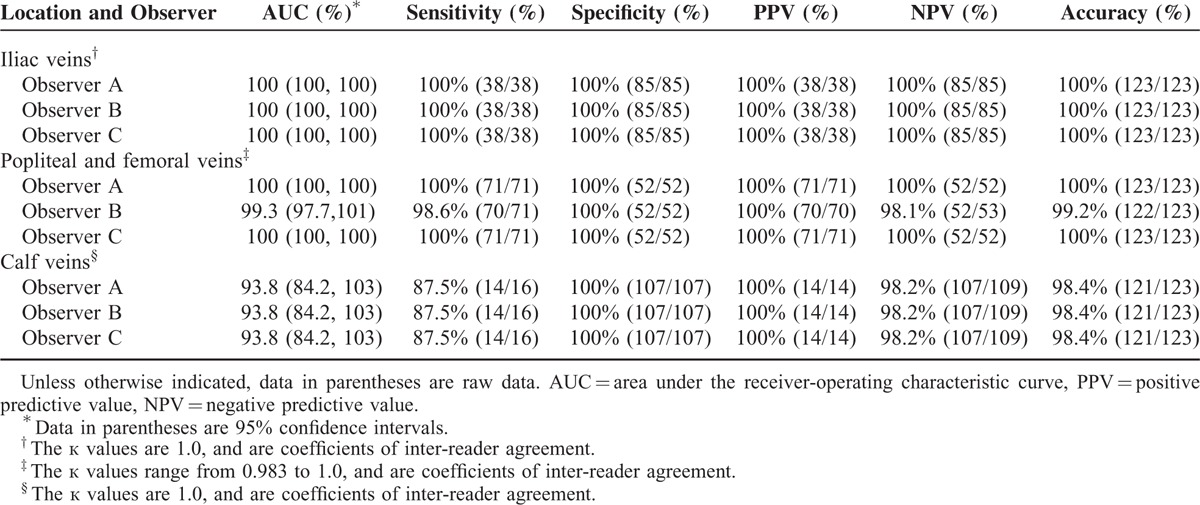
Diagnostic Performance of Combined Direct and Indirect CTV in Location-based Evaluation

**TABLE 4:**
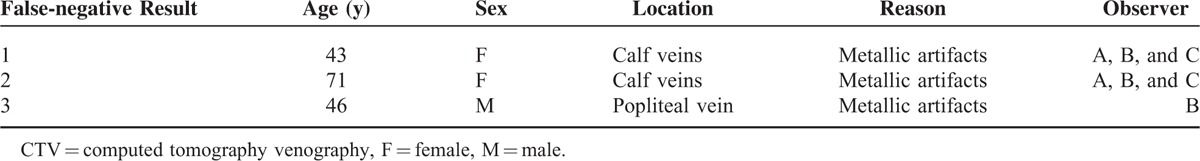
Details of all False-negative Results With Combined Direct and Indirect CTV

**FIGURE 2:**
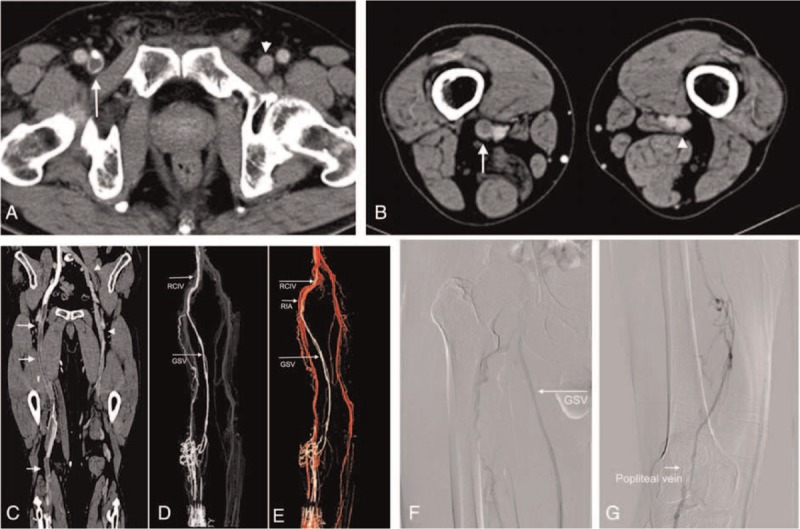
A 70-year-old man with right lower limb swelling for 7 days who was examined with combined CTV. Cross-sectional images show “filling defect” sign (white arrow) in the proximal (A) and distal (B) femoral vein, indicating acute thrombosis. Note the left lower limb deep veins that are simultaneously revealed. The degree of enhancement of a true thrombus was less than that of the opposite vein on the same level. An image obtained with curved planar reconstruction (C) shows extensive thrombosis in the femoral and popliteal veins (white arrows). Images reconstructed with maximum intensity projection (D) and volume rendering (E) show the vessel tree of the right limb. The contrast medium was mainly flowing through the right great saphenous vein (GSV) and the right common iliac vein (RCIV). Ascending venography (F, G) shows that the superficial femoral vein is not visible, indicating thrombotic occlusion.

**FIGURE 3:**
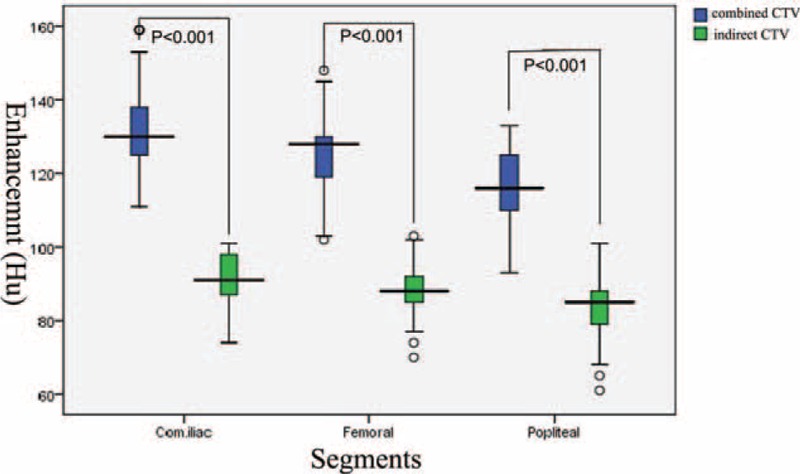
Comparison of venous enhancement (HU) (combined versus indirect CTV) in the common iliac vein, the proximal femoral vein, and the popliteal vein.

Combined CTV was also able to provide indirect CTV on the opposite limb (Figure 2), enabling comparison of both (combined CTV on the diseased limb versus indirect CTV on the opposite limb) on the same image. All anatomical levels of the pelvic and lower limb venous system were clearly depicted on combined CTV. The image quality of combined CTV was superior to that of indirect CTV. In the negative control group, we measured the enhancement of the common iliac vein, the proximal femoral vein, and the popliteal vein bilaterally. On the limb with combined CTV, the degree of enhancement was higher than that measured on the limb with indirect CTV at the same vein segment (*P* < 0.001) (Figure 3).

## DISCUSSION

We used patient- and location-based evaluations and compared the results validated with DSA and US. We showed high accuracy and specificity of >95% when combined direct and indirect CTV was used to detect LEDVT. Combined direct and indirect CTV images also provided an accurate 3D representation of the whole venous system of the lower limb with a realistic 3D model of the limbs, allowing a comprehensive morphological study of the deep veins. These results indicate that the current technology for combined direct and indirect CTV can be safely used in lower limbs in the diagnostic work-up of patients with LEDVT. To the best of our knowledge, this study is the first to investigate combined direct and indirect CTV in the detection of LEDVT.

Traditional phlebography is no longer considered the gold standard for morphofunctional examination of the venous network of the lower limbs. The reason for this is because, in the majority of cases, US provides not only anatomical data, but most importantly, a complete hemodynamic evaluation.^10^ Therefore, in the present study, we used a combined examination of phlebography plus US as the gold reference. However, the anatomy of the venous system of the lower extremities is extremely complex and variable. In complicated cases, global and morphologically accurate assessment of the vein tree cannot be exclusively assessed with a single diagnostic modality.^10,11^ US can have difficulty in detecting proximal DVT in the pelvis because of excess bowel gas, a large body habitus, an in situ inferior vena cava filter, and postsurgical abdomen or acute abdomen.^10,12,13^ Additionally, providing clinicians with 3D anatomical information on concomitant venous diseases is always a challenge with US (eg, proximal vein obstruction). With the advances made in recent years, CTV can provide such information, thus helping in determining treatment strategies for patients with LEDVT.

The advantages of CTV include the scanning speed of CT and the excellent density contrast of images, as well as 3D images rendered by post-processing techniques. Traditionally, indirect CTV of the lower limbs has been used as a part of the work-up of venous thromboembolism. Indirect CTV is concurrently performed with CT pulmonary angiography, requires no additional consumption of contrast medium, and adds an incremental benefit of 26% to 28% in the diagnosis.^14–19^ The sensitivity and specificity of indirect CTV in the detection of DVT are considered comparable with those of lower-limb US.^10,20,21^ Furthermore, the use of CTV has been emphasized for detection of isolated abdominal or pelvic vein thrombosis.^20^ However, indirect CTV might have inadequate contrast opacification of the veins when the scanning parameters and timing of venous phase acquisition are inappropriately used, accounting for suboptimal or nondiagnostic CTV studies in 3.1% to 15.2% of cases.^14,15^ Another limitation of indirect CTV is that contrast opacification of the veins might fail to reach a level that renders high quality of 3D images.

Direct CTV has one notable and outstanding advantage as follows. With postprocessing techniques, direct CTV can render 3D modeling of the venous system. Therefore, a comprehensive anatomical evaluation of the whole vessel tree can be made.^10^ This might be vital for planning before performing surgery or endovascular treatment. By checking the original slices, direct CTV can also be used to investigate LEDVT, as well as its potential underlying obstruction in the abdominal or pelvic vein. The main problem of direct CTV is the possible lack of injection of some veins, mimicking a “filling defect” sign, and this may result in a false-positive diagnosis of DVT.^10^ This situation may occur in cases of huge or highly located varicose veins, popliteal fossa veins, and calf veins.^10,22^ An improper bolus timing of contrast medium, usually an injection time that is too short, and inappropriate compression on a limb may contribute to this lack of injection of the territory of interest.^10,22^ Artifacts may also occur at the confluence of the great saphenous vein and the femoral vein, where usually there is turbulent flow. This might induce an imbalanced concoction of contrast medium and blood and manifestation of hypodense or hyperdense streaks on CTV.

In the present study, we designed a technique called combined CTV to investigate the venous system of the lower limbs and LEDVT. Combined CTV could theoretically overcome some flaws either belonging to indirect or to direct CTV. Apart from 2 injections of contrast medium, the scanning technique for combined CTV is the same as that for direct or indirect CTV. As mentioned above, the patients in our study had combined CTV performed on the diseased limb and indirect CTV performed on the opposite side. Therefore, we were able to compare direct and indirect CTV on the same image. First, a localized “filling defect” is mostly likely to be a true thrombus, if the degree of enhancement on combined CTV is inferior to the attenuation measured on the same level of the vein on the opposite limb. The “filling defect” might otherwise be a forgery if it has an enhancement level higher than the opposite side. Second, combined CTV can render 3D modeling of the venous system, which may help in preoperational planning. Third, combined CTV had an excellent diagnostic performance in our study. Only metallic artifacts in a few cases caused misdiagnosis of DVT. No false-positive diagnosis was made in the present study.

Combined CTV requires more contrast medium than that of indirect or direct CTV, which may increase the renal burden, especially in case of renal dysfunction. The biggest concern that has been raised is an additional radiation dose that is applied to patients using spiral acquisition protocols.^16,21^ However, a scanning protocol with a low radiation dose may lead to false-negative interpretation.^21,23^ As a preliminary study, we did not study the relationship between the imaging quality of combined CTV and the radiation dose. We believe that the radiation dose could be reduced to an acceptable level without sacrificing any imaging quality. However, the scanning protocol of combined CTV should be optimized in future studies.

There are some limitations of the present study. First, this was a single-center study comprising a relatively small sample. Therefore, the results from the present study might be limited when expanded to large-scale use. Second, because of the rising popularity of US in investigating LEDVT, it must be more reasonable to invest combined CTV with significance just on complicated cases, such as in the case of proximal DVT secondary to May–Thurner syndrome. Third, concerning harm from radiation, magnetic resonance venography may represent another option.^23^ Finally, we could not perform direct, indirect, and combined CTV on the same patient simultaneously because of ethical concerns. A case–control study should be conducted in the future to compare the 3 methods in terms of diagnostic performance, radiation dose, and contrast medium.

In conclusion, combined CTV shows high diagnostic accuracy in the detection of LEDVT, and this accuracy appears to be similar to that obtained with DSA and US. We recommend that combined CTV be used as a complimentary tool in the diagnostic work-up of patients with LEDVT, especially in complicated cases. This is because of the high degree of accuracy and sensitivity and excellent capability of 3D modeling of the venous system in combined CTV.
